# K284-6111 alleviates memory impairment and neuroinflammation in Tg2576 mice by inhibition of Chitinase-3-like 1 regulating ERK-dependent PTX3 pathway

**DOI:** 10.1186/s12974-020-02022-w

**Published:** 2020-11-22

**Authors:** Hyeon Joo Ham, Yong Sun Lee, Jaesuk Yun, Dong Ju Son, Hee Pom Lee, Sang-Bae Han, Jin Tae Hong

**Affiliations:** College of Pharmacy and Medical Research Center, Chungbuk National University, Osongsaengmyeong 1-ro, Osong-eup, Heungdeok-gu, Cheongju, Chungbuk 28160 Republic of Korea

**Keywords:** Alzheimer’s disease, Neuroinflammation, CHI3L1, ERK, NF-κB

## Abstract

**Background:**

Alzheimer’s disease (AD) is one of the most prevalent neurodegenerative disorders characterized by gradual memory loss and neuropsychiatric symptoms. We have previously demonstrated that the 2-({3-[2-(1-cyclohexene-1-yl)ethyl]-6,7-dimethoxy-4-oxo-3,4-dihydro-2-quinazolinyl}sulfanyl)-N-(4-ethylphenyl)butanamide (K284-6111), the inhibitor of CHI3L1, has the inhibitory effect on memory impairment in Αβ infusion mouse model and on LPS-induced neuroinflammation in the murine BV-2 microglia and primary cultured astrocyte.

**Methods:**

In the present study, we investigated the inhibitory effect of K284-6111 on memory dysfunction and neuroinflammation in Tg2576 transgenic mice, and a more detailed correlation of CHI3L1 and AD. To investigate the effects of K284-6111 on memory dysfunction, we administered K284-6111 (3 mg/kg, p.o.) daily for 4 weeks to Tg2576 mice, followed by behavioral tests of water maze test, probe test, and passive avoidance test.

**Results:**

Administration of K284-6111 alleviated memory impairment in Tg2576 mice and had the effect of reducing the accumulation of Aβ and neuroinflammatory responses in the mouse brain. K284-6111 treatment also selectively inactivated ERK and NF-κB pathways, which were activated when CHI3L1 was overexpressed, in the mouse brain and in BV-2 cells. Web-based gene network analysis and our results of gene expression level in BV-2 cells showed that CHI3L1 is closely correlated with PTX3. Our result revealed that knockdown of PTX3 has an inhibitory effect on the production of inflammatory proteins and cytokines, and on the phosphorylation of ERK and IκBα.

**Conclusion:**

These results suggest that K284-6111 could improve memory dysfunction by alleviating neuroinflammation through inhibiting CHI3L1 enhancing ERK-dependent PTX3 pathway.

## Background

Alzheimer’s disease (AD) is the most common neurodegenerative disease, with 46.8 million people currently suffering from AD worldwide, and the number is expected to reach 131.5 million by 2050 [1]. AD is generally characterized by reduced cognitive function, including memory loss, but it is also characterized by behavioral or psychological symptoms such as anxiety, depression, and delusions [2]. Despite decades of research, AD is still one of the major human medical challenges that are incurable [3]. Currently, there are six FDA-approved drugs for treating AD, but none of them stop the progression or provide any fundamental treatment for AD [4].

The pathological features of AD are accumulation of β-amyloid (Aβ) plaques, neurofibrillary tangles, cerebral atrophy, and neuroinflammation [5, 6] It is known that neuroinflammatory responses occur in the AD brain, such as changes in morphology and distribution of microglia and astrocytes, and increased expression of inflammatory mediators [7]. One of the major factors involved in neuroinflammation in the central nervous system (CNS) is the activation of microglia, which is the major cell type having a role of immune function in CNS whenever injure occurs [8, 9]. From genome-wide association studies (GWAS), a lot of single nucleotide polymorphisms (SNPs), which have been shown to be associated with differential AD risk, are exclusively or most highly expressed in microglia [10]. Microglia have two types of activation phenotype within the inflammatory environment: the traditional activation (M1) phenotype and the alternative activation (M2) phenotype [8, 9]. The M1 phenotype is the pro-inflammatory microglia, the main characteristic of which is the production of pro-inflammatory mediators such as pro-inflammatory cytokines including tumor necrosis factor (TNF)-α, interleukin (IL)-1β, IL-6, and inflammatory proteins [11, 12]. On the other hand, the M2 phenotype is the anti-inflammatory microglia, the main characteristic of which is the production of anti-inflammatory mediators such as anti-inflammatory cytokines including arginase-1 (Arg1), transforming growth factor β1 (TGFβ1), and mannose receptor C-type 1 (MRC1), and immunosuppressive proteins [11, 12]. Previous studies have shown that inflammatory damage due to excessive M1 microglia activation and dysfunction of M2 microglia further develop AD [13]. In the APP/PS1 AD mouse model, markers of the M1 microglia phenotype, such as CD36, CD14, CD11c, MHC-II, and iNOS, in particular, strong expression of CD11b and TREM2, and highly activated phenotypes surrounding synaptic clefts were increased [14]. In the brain of AD patients, apoptotic and pro-inflammatory signaling including M1 microglia phenotypes such as IFN-γ, and IL-1β are upregulated [8].

Chitinase-3-like 1 (CHI3L1), which is expressed in various cell types including epithelial cells, smooth muscle cells, macrophages, and neutrophils, is a glycoprotein that binds to chitin but lacks chitin hydrolase activity [15, 16]. There is growing evidence showing that CHI3L1 plays a critical role in inflammation, proliferation, and angiogenesis, and is associated with a lot of diseases including rheumatoid arthritis, liver fibrosis, inflammatory bowel disease, and neurological diseases [17, 18]. Specifically, CHI3L1 is up-regulated in various diseases characterized by chronic inflammation [19, 20]. It is known that pro-inflammatory cytokines such as IFN-γ, TNF-α, IL-1β, and IL-6 are involved in the expression of CHI3L1 [21]. The excessive increase in CHI3L1 can have unpredictable pathological effects by initiating and persisting chronic inflammation [22]. Several clinical studies reported that the elevation of CHI3L1 was observed in patients suffering from a wide range of diseases: cancer, metabolic, and neurological diseases [23–28]. Recent studies have shown a significant increase in CHI3L1 in cerebrospinal fluid in AD patients, correlating with widely accepted biomarkers of AD such as tau proteins or Aβ [29].

Pentraxin-3 (PTX3) is the prototypic member of the long pentraxin family and is expressed in a variety of cell types, including monocytes, macrophages, dendritic cells, adipocyte, fibroblasts, vascular smooth muscle cells, and endothelial cells [30]. PTX3 can be upregulated by lipopolysaccharide (LPS), IL-1β, IL-10, and TNF-α [31]. Zhao et al. reported that knockdown of PTX3 inhibited the production of nitric oxide and the expression of iNOS in HUVEC cells [32]. Ko et al. reported that PTX3 secretion exacerbates neuronal cell death; therefore, PTX3 secretion could worsen AD [33]. Using the web-based gene network analysis, we found that PTX3 was associated with CHI3L1 (Fig. 7a). However, the involvement of PTX3 in the CHI3L1-mediated neuroinflammation and the role of PTX3 in microglia polarization still unclear.

In our previous study, we found that the 2-({3-[2-(1-cyclohexene-1-yl)ethyl]-6,7-dimethoxy-4-oxo-3,4-dihydro-2-quinazolinyl}sulfanyl)-N-(4-ethylphenyl)butanamide (K284-6111) alleviated memory dysfunction and neuroinflammation by inhibiting CHI3L1 in Αβ infusion AD mouse model [34]. However, studies of the detailed mechanisms of K284-6111 that inhibits neuroinflammation and memory impairment by inhibition of CHI3L1 function have not been performed yet. In the present study, we investigated that K284-6111 could exert these inhibitory effects in another AD mouse model, Tg2576 transgenic mice, and the more detailed mechanisms of K284-6111 action.

## Methods

### Materials

The 2-({3-[2-(1-cyclohexene-1-yl)ethyl]-6,7-dimethoxy-4-oxo-3,4-dihydro-2-quinazolinyl}sulfanyl)-N-(4-ethylphenyl)butanamide (K284-6111) was synthesized by Professor Jae-Kyung Jung, a specialist in pharmaceutical organic chemistry. The K284-6111 was dissolved in Dimethyl sulfoxide (DMSO; final concentration of 100 μM) and stored at −20 °C until use. The Aβ_1-42_ was purchased from Sigma Aldrich (St. Louis, MO, USA). The U0126, SP600125, SB203580, and Bay 11-7082 were purchased from Sigma Aldrich (St. Louis, MO, USA).

### Animal and treatment

Twelve-month-old Tg2576 mice were maintained and handled in accordance with the guidelines for animal experiments of the institutional animal care and use committee of the Laboratory Animal Research Center at Chungbuk National University, Korea (ethics approval No. CBNUA-1329-19-01). All efforts were made to minimize animal suffering and to reduce the number of animals used. All mice were housed in 4-mouse cages with automatic temperature control (21–25 °C) at relative humidity levels of 45 to 65% with a 12-hour light-dark cycle. Food and water were provided ad libitum. Tg2576 mice harboring human APP695 with Swedish double mutation (hAPP; HuAPP695; K670N/M671L) were purchased from Taconic Farms (Germantown, NY, USA), and the strain was maintained in the animal laboratory at Chungbuk National University. Tg2576 mice were randomly divided into two groups: (I) the control vehicle-treated group (*n* = 12, ♂6, ♀6) and (II) the K284-6111 (3 mg/kg)-treated group (*n* = 13, ♂6, ♀7). The K284-6111 was administered by oral gavage for 4 weeks daily. The K284-6111 solution to be administered to the mice was prepared by dissolving K284-6111 stock (100 μM) dissolved in DMSO in saline to the dose of 3 mg/kg. Control mice were alternatively given an equal volume of vehicle. The behavioral tests of learning and memory capacity were assessed using the water maze, probe, and passive avoidance tests. Mice were sacrificed after behavioral tests by CO_2_ asphyxiation.

### Water maze test

The water maze test is a commonly accepted method for assessing cognitive function, and thus we performed the water maze test to measure memory capacity according to the modified protocol of the Morris water maze [35]. Maze testing was carried out by the SMART-CS (Panlab, Barcelona, Spain) program and equipment. A circular plastic pool (height 35 cm, diameter 100 cm) was filled with water made opaque with skim milk kept at 22–25 °C. An escape platform (height 14.5 cm, diameter 4.5 cm) was submerged 1–1.5 cm below the surface of the water in position. Testing trials were performed on a single platform and at two rotational starting positions. Each trial lasted for 60 s or ended as soon as the mouse reached the submerged platform. After the testing trial, the mice were allowed to remain on the platform for 120 s and were then returned to their cage. Escape latency and escape distance of each mouse were monitored by a camera above the center of the pool connected to a SMART-LD program (Panlab, Barcelona, Spain). A quiet environment, consistent lighting, constant water temperature, and a fixed spatial frame were maintained throughout the experimental period.

### Probe test

To assess memory retention, a probe test was performed 24 h after the water maze test. The platform was removed from the pool which was used in the water maze test, and the mice were allowed to swim freely. The swimming pattern of each mouse was monitored and recorded for 60 s using the SMART-LD program (Panlab, Barcelona, Spain). Retained spatial memory was estimated by the time spent in the target quadrant area.

### Passive avoidance performance test

The passive avoidance test is generally accepted as a simple method for testing memory. The passive avoidance response was determined using a “step-through” apparatus (Med Associates Inc., Vermont, USA) that is divided into an illuminated compartment and a dark compartment (each 20.3 × 15.9 × 21.3 cm) adjoining each other through a small gate with a grid floor, 3.175 mm stainless steel rods set 8 mm apart. A training trial was performed 2 days after the probe test. For the training trial, the mice were placed in the illuminated compartment facing away from the dark compartment. When the mice moved completely into the dark compartment, it received an electric shock (0.45 mA, 3 s duration). Then, the mice were returned to their cage. One day after the training trial, the mice were placed in the illuminated compartment and the latency to enter the dark compartment defined as “retention” was measured. The time taken for the mice entered into the dark compartment was recorded and described as step-through latency. The cut-off time limit of the retention trials was set at 3 min.

### Collection and preservation of brain tissues

After the completion of all the behavioral tests, the mice were perfused with PBS with heparin under inhaled CO_2_ anesthetization. The brain was immediately removed from the skull of the mouse, separated into the left and right brain, and randomly allocated either for protein or RNA analysis or fixation in a 10% formalin solution for 3 days at room temperature. Hippocampal tissue for protein or RNA analysis was immediately isolated after perfusion, divided vertically in half, and stored at −80 ° C until use.

### Thioflavin S staining

The brain fixed in a 10% formalin solution was embedded in paraffin wax, and then the brain was cut into sections 5-μm-thick slices. Thioflavin S staining was performed as described previously [4]. The sections were mounted in a mounting medium (Vectashield® mounting medium for fluorescence with DAPI; Vector Laboratories, Burlingame, CA, USA). The thioflavin S staining was examined using a confocal fluorescence microscope (K1-Fluo; Nanoscope systems, Daejeon, Korea) (×50 and ×200).

### Assay of β-secretase activities

β-secretase activity in the mice brains was determined using a commercially available β-secretase activity kit (Abcam, Inc., Cambridge, MA, USA). Solubilized membranes were extracted from hippocampus tissues using β-secretase extraction buffer, incubated on ice for 1 h, and centrifuged at 5000×*g* for 10 min at 4 °C. The supernatant was collected. A total of 50 μL of the sample (total protein 100 μg) or blank (β-secretase extraction buffer 50 μL) was added to each well (used 96-well plate) followed by 50 μL of 2X reaction buffer and 2 μL of β-secretase substrate incubated in the dark at 37 °C for 1 h. Fluorescence was read at excitation and emission wavelengths of 335 and 495 nm, respectively, using a fluorescence spectrometer (Gemini EM; Molecular Devices, CA, USA).

### ELISA assay

Cytokine levels (TNF-α, IL-1β, and IL-6) were measured by ELISA kits purchased from KOMA Biotech (Seoul, Korea) following the manufacturer’s protocol. CHI3L1 level was measured by ELISA kits purchased from R&D Systems (Minneapolis, MN, USA) following the manufacturer’s protocol.

### Western blot analysis

Homogenized brain hippocampus tissues were lysed by protein extraction solution (PRO-PREP, iNtRON, Kyungki-do, Korea) and the total protein concentration was determined using the Bradford reagent (Bio-Rad, Hercules, CA, USA). A total of 40 μg of extracted protein were separated by SDS/PAGE and transferred to Immobilon^Ⓡ^ PVDF membranes (Millipore, Bedford, MA, USA). The membrane was blocked with 5% BSA in Tris-buffered saline containing 0.05% Tween-20 (TBST) for 1 h at room temperature, followed by incubation with specific primary antibodies overnight at 4 °C. The membranes were washed with TBST and incubated with diluted HRP-conjugated secondary antibodies for 1 h at room temperature. After washes, binding of antibodies to the PVDF membrane was detected using the Immobilon Western Chemiluminescent HRP Substrate (Millipore, Bedford, MA, USA). The band intensities were measured using the Fusion FX 7 image acquisition system (Vilber Lourmat, Eberhardzell, Germany) and quantified using Image J software. To detect target proteins, specific primary antibodies against iNOS, IBA-1, GFAP, APP, and BACE1 (1:1000; Abcam, Inc., Cambridge, UK), COX-2 (1:1000; Novus Biologicals, Inc., CO, USA), ERK 1/2, p-ERK 1/2, JNK, p38, p-p38, IκBα, and p-IκBα (1:000; Cell signaling Technology, Inc., MA, USA), p-JNK and β-actin (1:200; Santa Cruz Biotechnology Inc., Santa Cruz, CA, USA), and PTX3 (1:1000; Invitrogen, Waltham, MA, USA) were used. The corresponding conjugated secondary antibodies such as anti-mouse, anti-rabbit, and anti-goat were purchased from Abcam (Cambridge, UK).

### Immunohistochemistry

The brain fixed in a 10% formalin solution was embedded in paraffin wax, and then the brain was cut into sections 5-μm-thick slices. Immunohistochemistry was performed as described previously [4]. To detect target proteins, specific antibodies against GFAP, IBA-1, iNOS (1:250; Abcam, Inc., Cambridge, MA, USA), and COX-2 (1:100, Novus Biologicals, Inc., CO, USA) were used. Brain sections were visualized by chromogen diaminobenzidine (Vector Laboratories, Burlingame, CA, USA). Finally, brain sections were mounted with mounting medium Cytoseal XYL (Thermo Scientific, Waltham, MA, USA) and evaluated on a light microscope (Microscope Axio Imager. A2; Carl Zeiss, Oberkochen, Germany; ×50 and ×200).

### Quantitative real-time PCR

The mRNA level was measured by quantitative real-time polymerase chain reaction (qRT-PCR). Total RNA was extracted using RiboEX (Geneall biotechnology, Seoul, Korea) from hippocampus tissue and cDNA was synthesized using high-capacity cDNA reverse transcription kit (Thermo Scientific, Waltham, MA, USA). Quantitative real-time PCR was performed on a 7500 real-time PCR system (Applied Biosystems, Foster City, CA, USA) for custom-designed primers and β-actin was used for house-keeping control using HiPi real-time PCR SYBR green master mix (ELPIS biotech, Daejeon, Korea). Cycling conditions consisted of an initial denaturation step of 3 min at 94 °C, a denaturation step of 30 s at 94 °C, an annealing step of 30 s at 60 °C, and an extension step of a minute at 72 °C followed by 40 cycles. The values obtained for the target gene expression were normalized to β-actin and quantified relative to the expression in control samples. Each sample was run with the following primer pairs shown in the supplementary material (Supplementary Table S1).

### BV-2 microglial cell culture

Microglial BV-2 cells were obtained from the American Type Culture Collection (Rockville, Maryland, United States). Microglial BV-2 cells were maintained with serum-supplemented culture media of DMEM supplemented with FBS (10%) and antibiotics (100 units/mL). The microglial BV-2 were incubated in the culture medium in a humidified incubator at 37 °C and 5% CO_2_. The cultured cells were treated with several concentrations (0.5, 1, 2 μM) of K284 -6111 or with U0126, SP600125, SB203580, and Bay 11-7082, 2 h before Aβ (5 μM) treatment or 24 h after transfection. The cells were harvested after 24 h.

### Transfection

BV-2 cells were transiently transfected with siRNA (20 nM/well/6-well plate) or using the Lipofectamine® RNAiMAX transfection reagent in Opti-MEM, according to the manufacturer’s specification (Invitrogen, Waltham, MA, USA). BV-2 cells were transiently transfected with pcDNA3.1(+)-6 × Myc-CHI3L1 vector or control vector using the Lipofectamine® 3000 transfection reagent in OPTI-MEM, according to the manufacturer’s specification (Invitrogen, Waltham, MA, USA). Negative control (NC), PTX3 siRNA were purchased from OriGene Technologies, Inc. (Rockville, MD, USA). pcDNA3.1(+)-6 × Myc-CHI3L1 vector was cloned from Bionics (Seoul, Republic of Korea).

### Gene network analyses

The gene network of CHI3L1 was analyzed using the web-based analysis tool, String (https://string-db.org), based on the publicly available biological datasets.

### Statistical analysis

The data were statistically analyzed using the GraphPad Prism software (Version 4.03; GraphPad Software, Inc., San Diego, CA, USA). Data are presented as mean ± S.E.M. The group differences in all data were assessed by Student’s *t* test, one-way analysis of variance (ANOVA) followed by the Tukey multiple comparison test, or two-way ANOVA followed by Bonferroni’s post hoc test. A value of *p* < 0.05 was considered statistically significant. *Significantly different between the two groups (*p* < 0.05). **Significantly different between the two groups (*p* < 0.01). ***Significantly different between the two groups (*p* < 0.001).

## Results

### K284-6111 alleviates memory impairment in Tg2576 mice

**Fig. 1:**
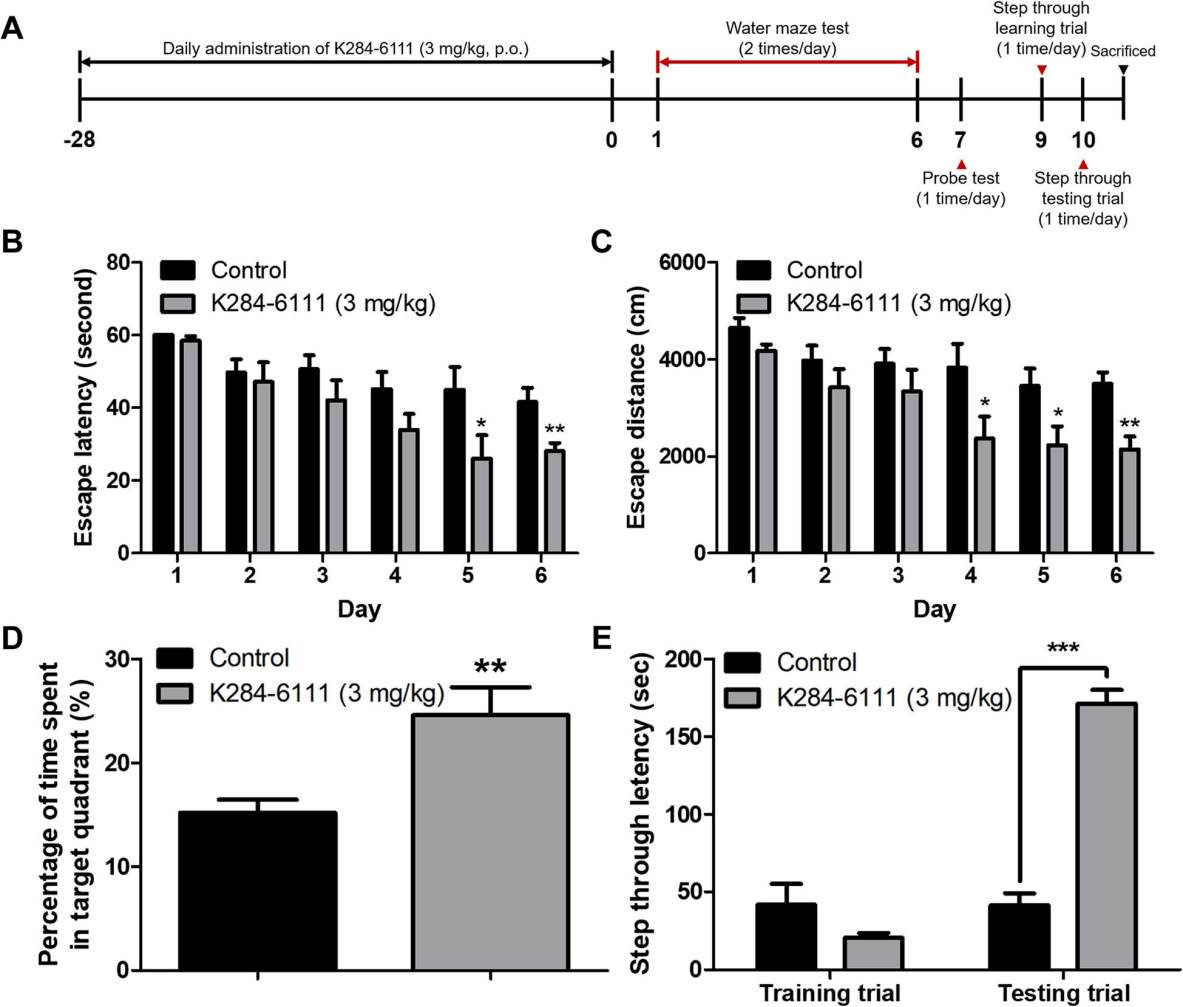
Effects of K284-6111 on memory impairment in Tg2576 mice. **a** A timeline has been described that demonstrates the administration of K284-6111 and the assessment of cognitive function in Tg2576 mice. To investigate the effect of K284-6111 on memory impairment, we carried out (**b**, **c**) the water maze test, (**d**) the probe test, and (**e**) the step-through type passive avoidance test. Memory and learning ability in Tg2576 were determined by the escape latencies (**b**, sec) and escape distance (**c**, cm) for 6 days, and time spent in the target quadrant (**d**, %) in the probe test

To investigate if there is a difference in CHI3L1 levels between Tg2576 and WT mice, the CHI3L1 levels in serum and brain were determined by ELISA assay. Both the serum CHI3L1 level and brain CHI3L1 level were significantly elevated in Tg2576 mice compared with WT mice (*n* = 7–8, *p <* 0.0001 and *p* < 0.0001, respectively; Supplementary Figure S1A, S1B). To assess the inhibitory effect of K284-6111 on memory impairment in Tg2576 AD mouse model, K284-6111 (3 mg/kg) was orally administered to Tg2576 mice daily for 4 weeks. After 4 weeks of administration, a series of behavioral tests were conducted to evaluate the memory of the Tg2576 mice (Fig. 1a). The spatial memory abilities in Tg2576 mice were assessed by the water maze test. On the final day of the water maze, the mean escape latency and swimming distance of the control group were about 41.6 ± 3.9 s and 3494 ± 238.7 cm, respectively. The K284-6111-treated group showed significantly decreased the mean escape latency and swimming distance compared to that of the control group, which were 28.1 ± 2.2 s and 2140 ± 272.4 cm, respectively (*n* = 12–13, time: F(5, 110) = 9.961, *p* < 0.0001, treatment: F(1, 110) = 1.551, *p* = 0.0046; Fig. 1b; time: F(5, 110) = 6.728, *p* < 0.0001, treatment: F(1, 110) = 18.05, *p* = 0.0003; Fig. 1c). In order to evaluate the effect of K284-6111 on memory consolidation in Tg2576 mice, the probe test was performed after removing the hidden platform in water the day after the final day of water maze testing. In the probe test, memory consolidation was determined by the percentage of the mean time spent in the target quadrant where the platform was located. The mean time spent in the target quadrant was significantly increased in the K284-6111-treated group (24.6 ± 2.7%) compared with that in the control group (15.2 ± 1.3%) (*n* = 12-13, *p* = 0.0073; Fig. 1d). To investigate the effect of K284-6111 on the memory retention ability of Tg2576 mice, the passive avoidance test was carried out. There was no significant difference between the two groups in the training trial, but the K284-6111-treated group showed higher an average step-through latency (171.2 ± 8.8 s) than that in the control group (41.2 ± 8.0 s) in the testing trial (*n* = 12–13, *p* < 0.0001; Fig. 1e).

### K284-6111 inhibits the accumulation of Aβ in Tg2576 mouse brain

**Fig. 2:**
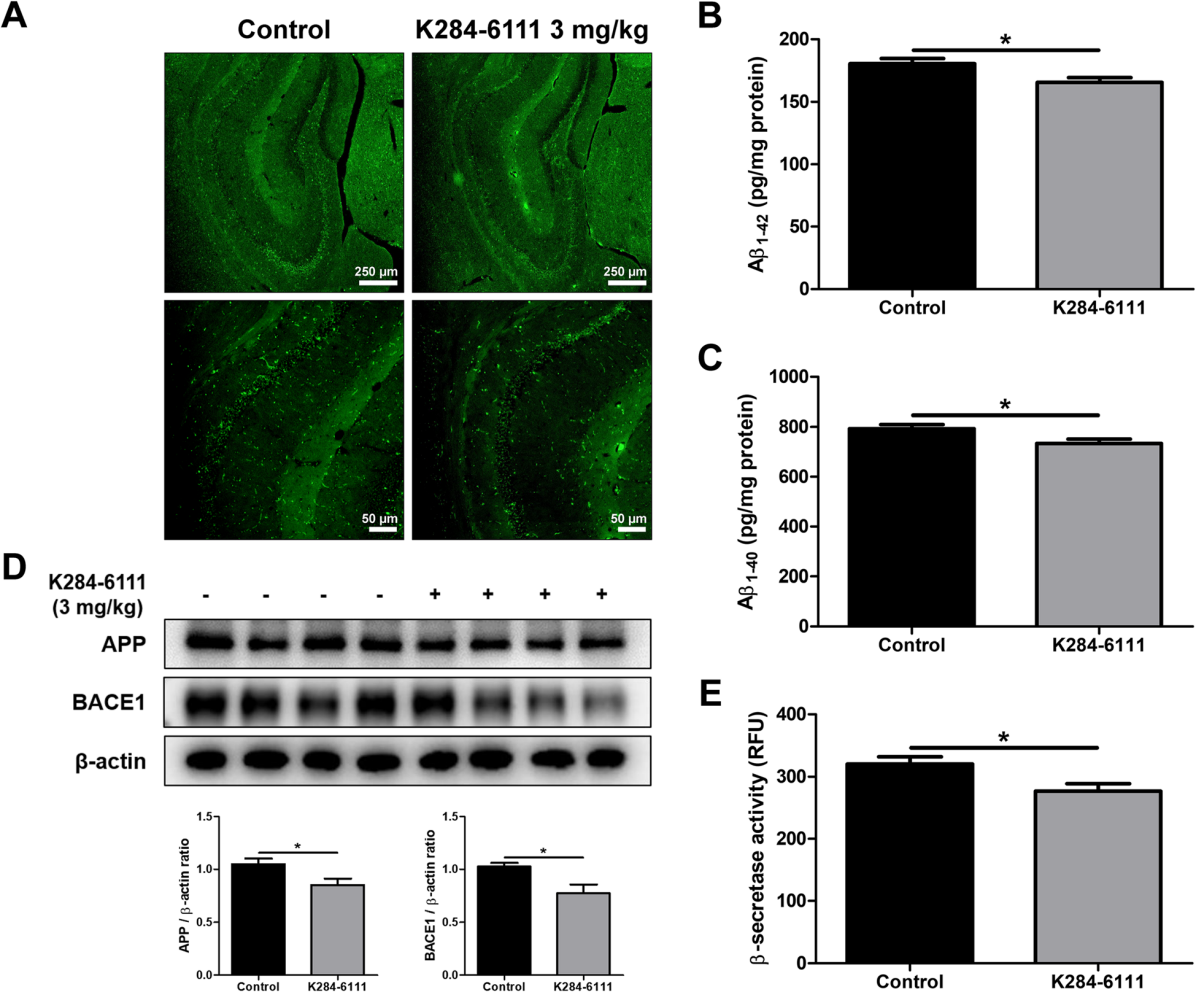
Effect of K284-6111 on the deposition of Aβ and amyloidogenic factors in Tg2576 mice brain. **a** The accumulation of Aβ plaques in the hippocampus was determined by Thioflavin S staining. **b**The levels of Aβ_1-42_ and (**c**) Aβ_1-40_ in Tg2576 mice brain were assessed using the specific ELISA kits. **d** The expression of APP and BACE1 were detected by Western blot. (**e**) The activity of β-secretase in mice brain was investigated by the β-secretase activity assay kit

The amyloid cascade hypothesis is by far the most well-known and accepted hypothesis of the causes of AD. According to this hypothesis, Αβ accumulation is highly associated with and is a major cause of AD. To investigate the effect of K284-6111 on the Aβ plaque accumulation in the brain of Tg2576 mice, thioflavin S staining was performed to stain β-sheet-rich structures of Aβ. The accumulation of Aβ plaques was reduced in the K284-6111-treated group compared with that in the control group (Fig. 2a). ELISA was performed to quantitatively measure the inhibitory effect of K284-6111 on Aβ accumulation in the brain of Tg2576 mice. The Aβ_1-42_ level in the mouse hippocampus was 180.4 ± 4.1 pg/mg of protein in the control group and 165.4 ± 3.9 pg/mg of protein in the K284-6111-treated group (*n* = 12-13, *p* = 0.0147; Fig. 2b). The Aβ_1-40_ level in the mouse hippocampus was 792.5 ± 16.7 pg/mg of protein in the control group and 733.7 ± 17.2 pg/mg of protein in the K284-6111-treated group (*n* = 12-13, *p* = 0.0231; Fig. 2c). Taken together, the K284-6111-treated group exhibits significantly lower Aβ levels than that of the control group. To determine how K284-6111 inhibits Aβ accumulation, we measured the levels of proteins and the activity of β-secretase involving in Aβ production. The administration of K284-6111 reduced the levels of APP and BACE1 in the brain of Tg2576 mice detected by the Western blot (Fig. 2d) and significantly reduced the β-secretase activity in the brain of Tg2576 mice (*n* = 12-13, *p* = 0.0178; Fig. 2e).

### Effect of K284-6111 against neuroinflammation in Tg2576 mouse brain

**Fig. 3:**
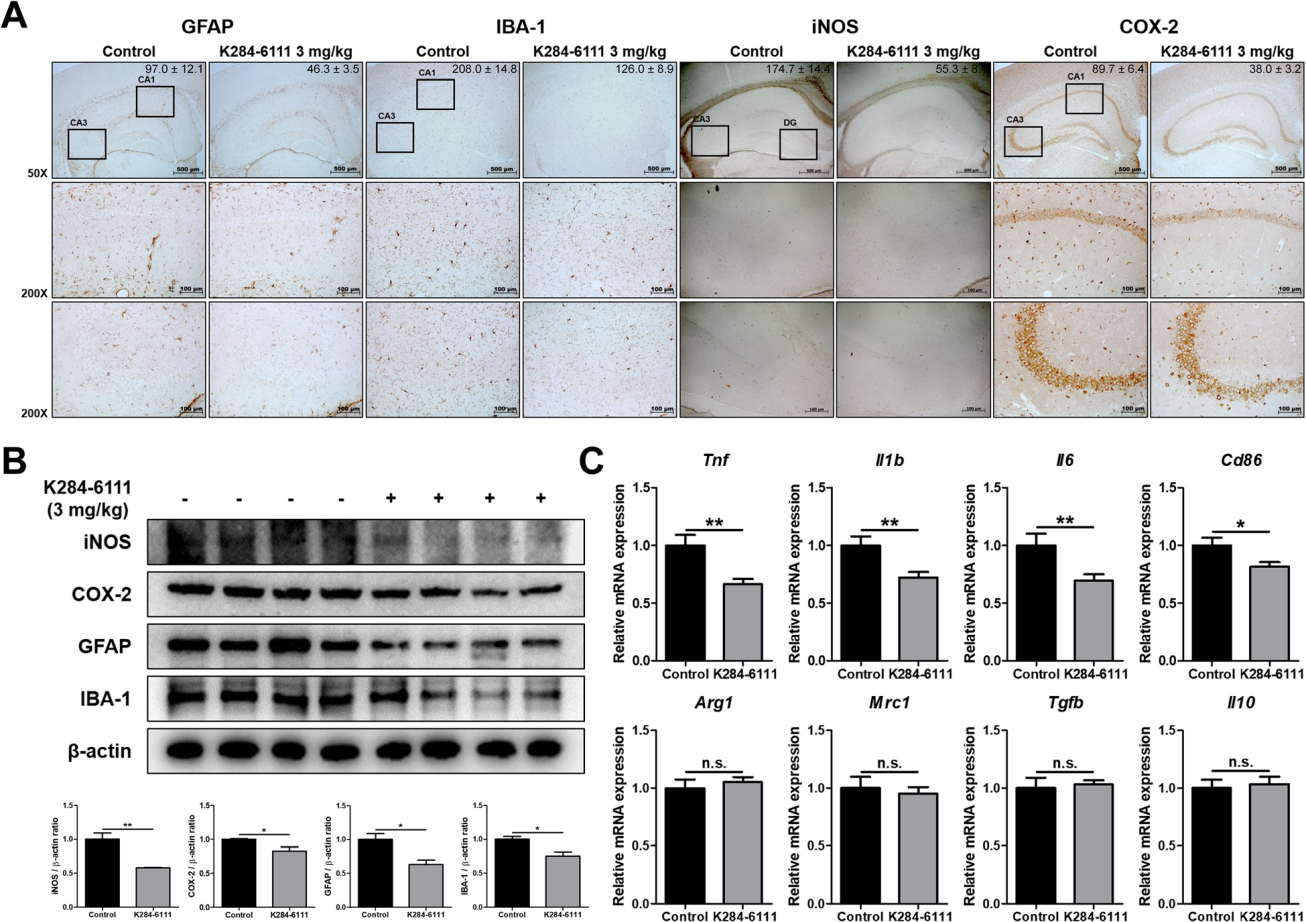
Inhibitory effect of K284-6111 on neuroinflammation in Tg2576 mice brain. **a** Expression of GFAP, IBA-1, iNOS, and COX-2 in Tg2576 mice hippocampus was determined by immunohistochemistry analysis. (**b**) Expression of iNOS, COX-2, GFAP, and IBA-1 was detected by Western blot. (**c**) The mRNA expression level of M1 microglia phenotype markers (*Tnf*, *Il1b*, *Il6*, and *Cd86*) and M2 microglia phenotype markers (*Arg1*, *Mrc1*, *Tgfb*, and *Il10*) were assessed by qRT-PCR

Increasing evidence suggests that the development of AD is accompanied by neuroinflammation, such as the activation of astrocytes or microglia. In order to investigate the effect of K284-6111 on neuroinflammation, immunohistochemistry, Western blot, and qRT-PCR were performed to determine changes in factors associated with neuroinflammation between the two groups. The number of GFAP (the marker of reactive astrocyte)-reactive cells and IBA-1 (the marker of activated microglia)-reactive cells was reduced in the K284-6111-treated group compared with that of the control group. The number of iNOS and COX-2-reactive cells involved in the neuroinflammatory response was reduced in the brain of the K284-6111-treated group (Fig. 3a). Consistent with immunohistochemistry results, the expression of GFAP, IBA-1, iNOS, and COX-2 in Western blot results also significantly decreased in the brain of the mouse treated with K284-6111 (Fig. 3b). There have been reports that the activation of microglia to excessive M1 phenotype or dysfunction of the M2 phenotype contributes to AD development [13]. To investigate the effect of K284-6111 on two phenotypes of activated microglia, M1 phenotype and M2 phenotype, which play a major role in neuroinflammatory conditions, the expression levels of the markers of M1 microglia (*Tnf*, *Il1b, Il6,* and *Cd86*) and the markers of M2 microglia (*Arg1, Mrc1, Tgfb,* and *Il10*) were determined by qRT-PCR. M1 microglia markers such as *Tnf*, *Il1b, Il6,* and *Cd86* were significantly decreased in the brain of the K284-6111-treated group, but M2 microglia markers such as *Arg1, Mrc1, Tgfb,* and *Il10* were hardly affected by the administration of K284-6111 (*n* = 10–12; *Tnf*: *p* = 0.0019; *Il1b*: *p* = 0.0046; *Il6*: *p* = 0.0097; *Cd86*: *p* = 0.0199; *Arg1*: *p* = 0.5442; *Mrc1*: *p* = 0.6494; *Tgfb*: *p* = 0.7419; *Il10*: *p* = 0.7462) (Fig. 3c).

### Effect of K284-6111 against neuroinflammation in murine microglial BV-2 cells

**Fig. 4:**
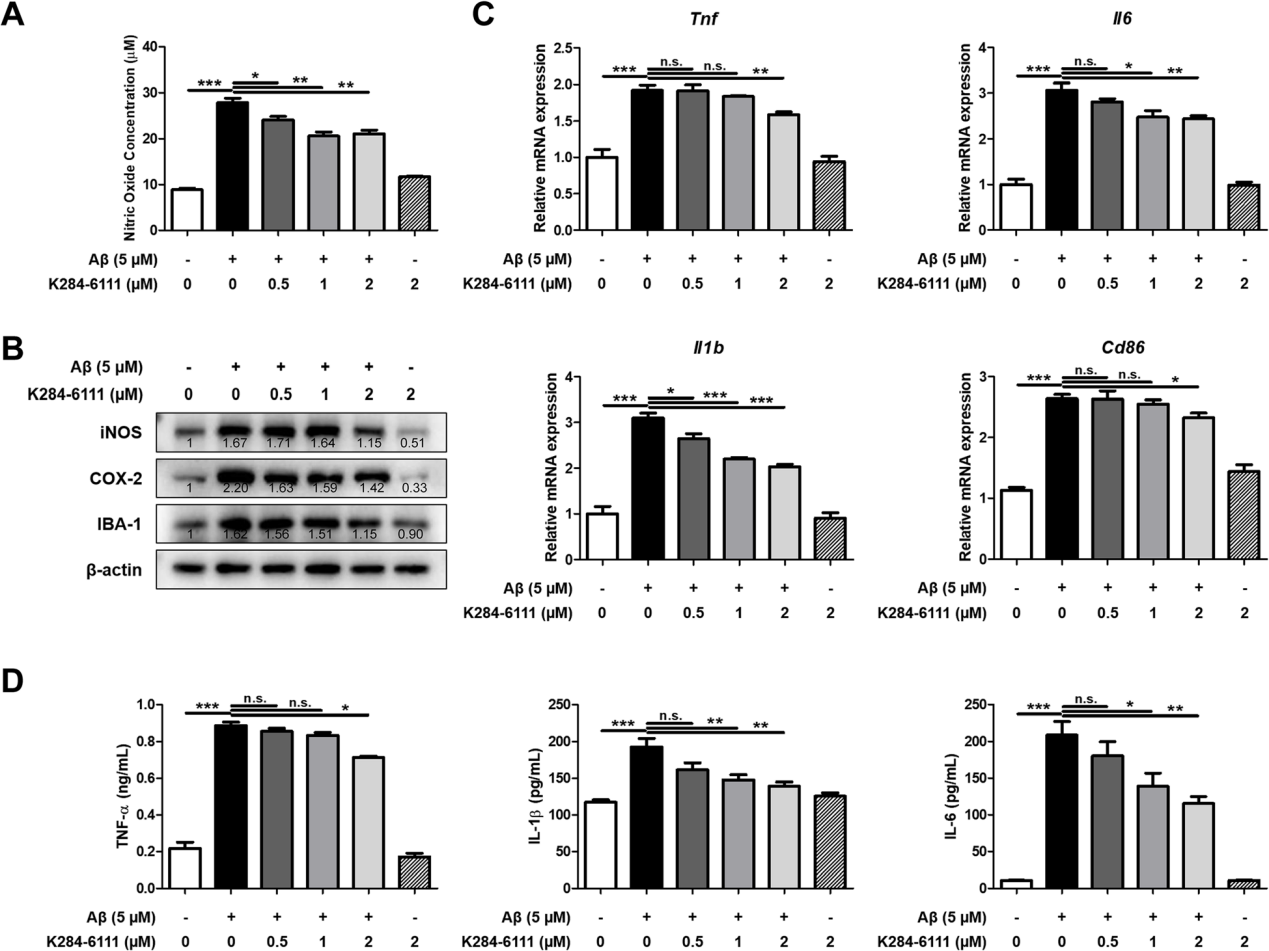
Inhibitory effect of K284-6111 on Aβ-induced neuroinflammation in BV-2 cells**. B**V-2 cells were treated with Aβ (5 μM) and K284-6111 (0.5, 1, and 2 μM). **a** The inhibitory effect of K284-6111 on nitric oxide production was determined using a nitric oxide assay kit in BV-2 cells. **b** Expression of iNOS, COX-2, and IBA-1 was detected by Western blot. **c** The mRNA expression level of pro-inflammatory cytokines (*Tnf*, *Il1b*, and *Il6*) and M1 microglia phenotype marker (*Cd86*) were assessed by qRT-PCR. (D) The production levels of TNF-α, IL-1β, and IL-6 were assessed using the specific ELISA kits

Activation of microglia is considered one of the major factors involved in neuroinflammation in AD. In order to explain the inhibitory effect of K284-6111, nitric oxide (NO) concentration and expression levels of inflammatory proteins and cytokines in BV-2 cells were measured. The NO concentration was elevated in the Aβ-treated group, and the NO concentration was decreased by the treatment of K284-6111 in a concentration-dependent manner (*n* = 4; F(5, 18) = 106.5, *p* < 0.0001) (Fig. 4a). The expression of iNOS, COX-2, and IBA-1 was also significantly increased by Aβ, and decreased in the K284-6111-treated groups (Fig. 4b). The mRNA expression level of pro-inflammatory cytokines such as *Tnf*, *Il1b,* and *Il6,* and *Cd86* as M1 microglia marker was increased in the Aβ-treated group, whereas their expression levels were significantly reduced by K284-6111 treatment in a concentration-dependent manner (*n* = 3-4; *Tnf*: F(5, 18) = 40.65, *p* < 0.0001; *Il1b*: F(5, 17) = 67.99, *p* < 0.0001; *Il6*: F(5, 17) = 75.28, *p* < 0.0001; *Cd86*: F(5, 18) = 54.97, *p* = 0.0729) (Fig. 4c). The level of pro-inflammatory cytokines such as TNF-α, IL-1β, and IL-6 was increased by Aβ treatment, whereas their levels were decreased by K284-6111 treatment (*n* = 3-4; TNF-α: F(5, 18) = 252.6, *p* < 0.0001; IL-1β: F(5, 17) = 11.51, *p* < 0.0001; IL-6: F(5, 18) = 37.35, *p* < 0.0001) (Fig. 4d).

### Inhibitory effect of K284-6111 on ERK and NF-κB signaling pathway

**Fig. 5:**
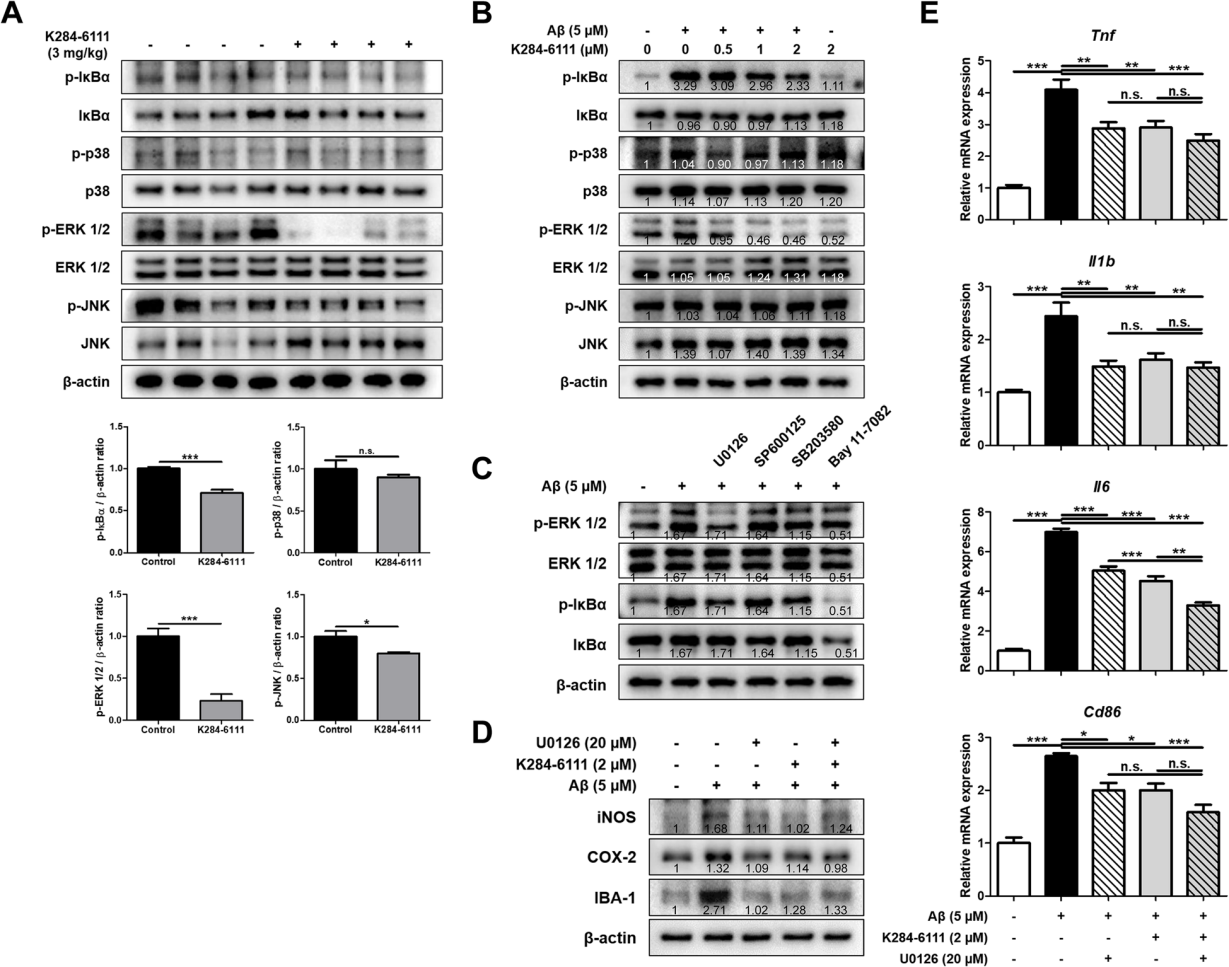
Inhibitory effect of K284-6111 on ERK and NF-κB pathway. **a** Level of p-IκBα, IκBα, p-p38, 38, p-ERK 1/2, ERK 1/2, p-JNK, and JNK were detected by Western blot in the Tg2576 mice brain. BV-2 cells were treated with Aβ (5 μM) and K284-6111 (0.5, 1, and 2 μM). **b** Level of p-IκBα, IκBα, p-p38, 38, p-ERK 1/2, ERK 1/2, p-JNK, and JNK were detected by Western blot. BV-2 cells were treated with Aβ (5 μM), U0126 (20 μM), SP600125 (20 μM), SB203580 (10 μM), and Bay 11-7082 (5 μM). (C) Level of p-ERK 1/2, ERK 1/2, p-IκBα, and IκBα were detected by Western blot. BV-2 cells were treated with Aβ (5 μM), K284-6111 (2 μM), and U0126 (20 μM). (D) Expression of iNOS, COX-2, and IBA-1 was detected by Western blot. (**e**) The mRNA expression level of pro-inflammatory cytokines (*Tnf*, *Il1b*, and *Il6*) and M1 microglia phenotype marker (*Cd86*) in BV-2 cells were assessed by qRT-PCR

To identify the signaling pathways involved in the anti-neuroinflammatory effects of K284-6111, the levels of NF-κB and mitogen-activated protein kinases (MAPK) signaling pathways known to be related to inflammation were determined using Western blot analysis in Tg2576 mouse brain and BV-2 microglial cells. Among the factors involved in these signaling pathways, the levels of p-IκBα, p-ERK1/2, and p-JNK were reduced in the brain of the K284-6111-treated group (Fig. 5a). In Aβ-induced BV-2 cells, the levels of p-IκBα and p-ERK were decreased in a concentration-dependent manner in the K284-6111-treated groups (Fig. 5b). To determine whether NF-κB and ERK signaling pathway are related to each other or to determine which of these two signals is the upper signal, ERK inhibitor (U0126; 20 μM), JNK inhibitor (SP600125; 20 μM), p38 inhibitor (SB203580; 10 μM), and NF-κB inhibitor (Bay 11-7082; 5 μM) were treated to BV-2 cells and the levels of p-ERK and p-IκBα were measured by Western blot. The level of p-IκBα was decreased with the ERK inhibitor (Fig. 5c). This result suggests that the ERK and NF-κB signals are implicated in the inhibitory effect of K284-6111 on neuroinflammation. To verify the combination effect of ERK inhibitor (U0126) and K284-6111 on neuroinflammation, the microglial BV-2 cells were treated with Aβ (5 μM), U0126 (20 μM), and K284-6111 (2 μM), and then the levels of M1 microglia markers and inflammatory proteins were measured. The intracellular levels of inflammatory proteins such as iNOS and COX-2, and the marker of microglia activation, IBA-1, increased by Aβ were reduced by U0126 or K284-6111 (Fig. 5d). However, when U0126 and K284-6111 were treated together, there was no better anti-inflammatory action than when U0126 or K284-6111 was treated respectively. The expression levels of *Tnf*, *Il1b, Il6,* and *Cd86*, which were increased by Aβ treatment, decreased when treated with U0126 or K284-6111. When U0126 and K284-6111 were treated together, the mRNA expression levels of *Tnf*, *Il1b,* and *Cd86* did not differ from co-treatment and single treatment, whereas the expression level of *Il6* showed a lower level when treated U0126 and K284-6111 together than single treatment (Fig. 5e).

### Inhibitory effect of K284-6111 on neuroinflammatory responses induced by CHI3L1

**Fig. 6:**
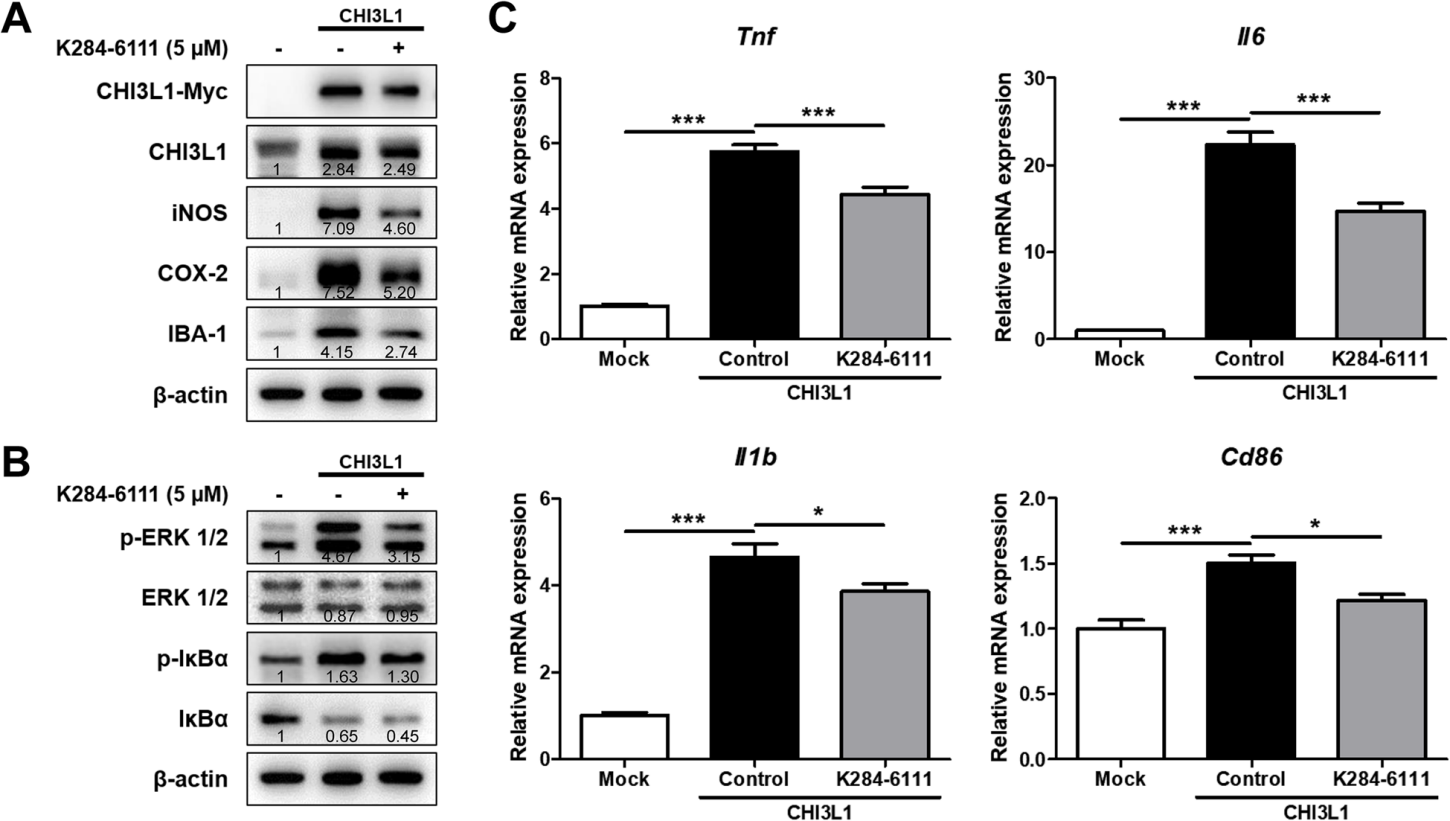
Inhibitory effect of K284-6111 on CHI3L1-induced neuroinflammation. BV-2 cells were transfected with CHI3L1 plasmid vector. After 24 hr, cells were treated with K284-6111 (5 μM). **a** Expression of iNOS, COX-2, and IBA-1 were detected by Western blot analysis using specific antibodies in BV-2 cells. **b** Level of p-ERK 1/2, ERK 1/2, p-IκBα, and IκBα were detected by Western blot. **c** The mRNA expression level of pro-inflammatory cytokines (*Tnf*, *Il1b*, and *Il6*) and M1 microglia phenotype marker (*Cd86*) in BV-2 cells were assessed by qRT-PCR

In our previous study, we demonstrated that K284-6111 directly binds to CHI3L1 using a computational docking study and that K284-6111 has an anti-inflammatory effect on the Aβ-infusion AD mouse model and BV-2 cells [34]. However, our previous study has not demonstrated whether CHI3L1 could cause neuroinflammation and whether K284-6111 could mitigate neuroinflammatory responses induced by CHI3L1. Thus, we transfected BV-2 cells with the CHI3L1 expression vector and then treated with K284-6111. BV-2 cells overexpressing CHI3L1 had higher levels of iNOS, COX-2, and IBA-1 and had higher levels of p-ERK and p-IκBα than that of normal cells (Fig. 6a, b). However, these levels elevated in CHI3L1 overexpressing BV-2 cells were decreased by the treatment of K284-6111. The expression of M1 microglia markers such as *Tnf*, *Il1b, Il6,* and *Cd86* was remarkably increased by CHI3L1 overexpression, however, significantly also decreased by treatment of K284-6111 (*n* = 6–8; *Tnf*: F(2, 21) = 186.5, *p* < 0.0001; *Il1b*: F(2, 19) = 108.2, *p* < 0.0001; *Il6*: F(2, 20) = 125.9, *p* < 0.0001; *Cd86*: F(2, 17) = 17.27, *p* = 0.0001) (Fig. 6c).

### K284-6111 inhibits PTX3-mediated neuroinflammation

**Fig. 7:**
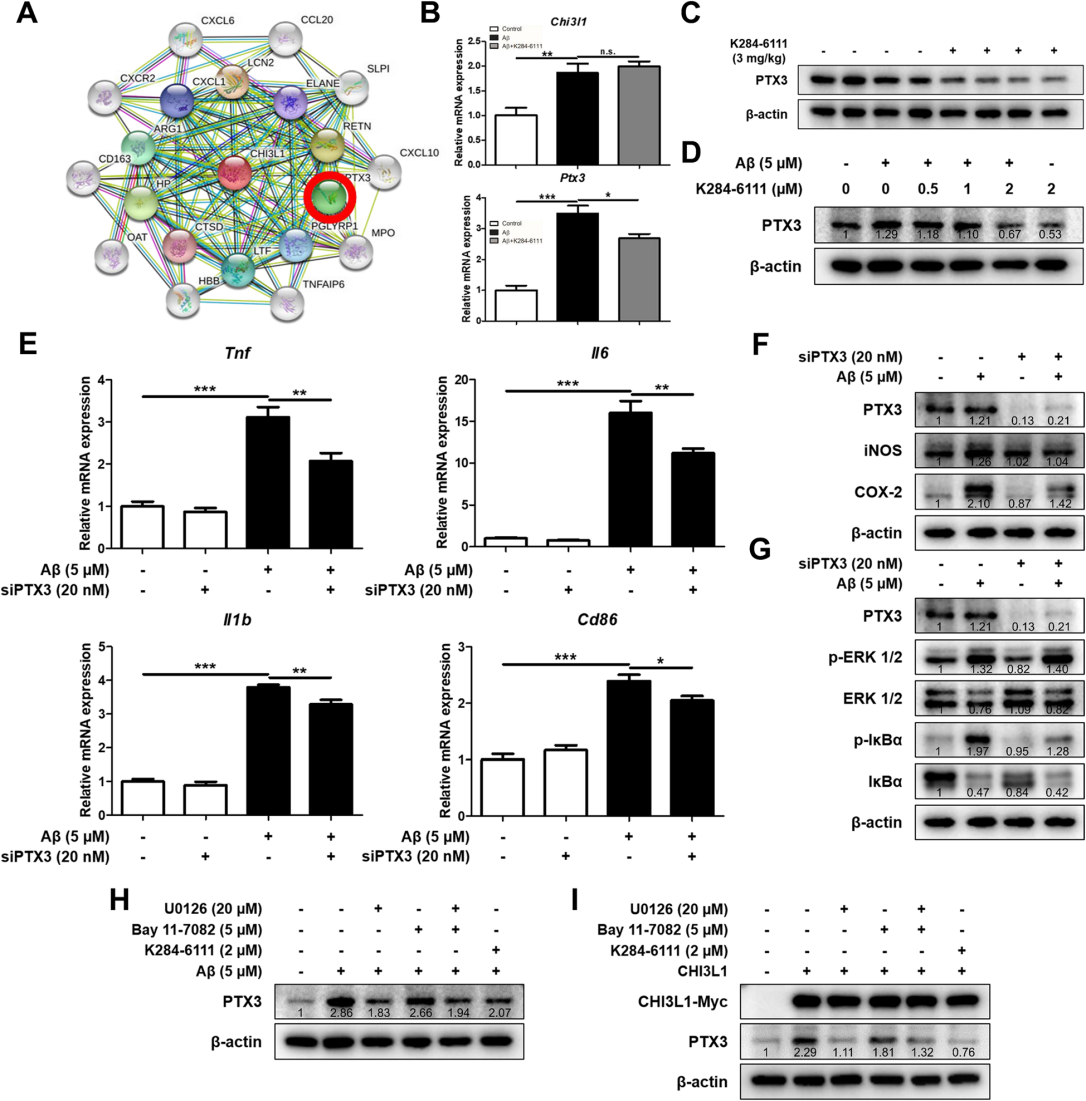
Effect of K284-6111 on PTX3-involved neuroinflammation. **a** Gene network analysis associated with CHI3L1 was carried out using the web-based analysis tool. BV-2 cells were treated with Aβ (5 μM) and K284-6111 (2 μM). (B) The mRNA expression level of *Chi3l1* and *Ptx3* in BV-2 cells was assessed by qRT-PCR. Expression of PTX3 was detected by Western blot (**c**) in the Tg2576 mice brain and (**d**) in the BV-2 cells. BV-2 cells were transfected with PTX3 siRNA (20 nM). After 24 hr, cells were treated with Aβ (5 μM). **e** The mRNA expression level of pro-inflammatory cytokines (*Tnf*, *Il1b*, and *Il6*) and M1 microglia phenotype marker (*Cd86*) in BV-2 cells were assessed by qRT-PCR. **f** Expression of iNOS and COX-2 was detected by Western blot. (G) Level of p-ERK 1/2, ERK 1/2, p-IκBα, and IκBα were detected by Western blot. BV-2 cells were treated with Aβ (5 μM), K284-6111 (2 μM), U0126 (20 μM), and Bay 11-7082 (5 μM). (H) Expression of PTX3 were detected by Western blot. BV-2 cells were transfected with CHI3L1 plasmid vector. After 24 hr, cells were treated with Aβ (5 μM), K284-6111 (2 μM), U0126 (20 μM), and Bay 11-7082 (5 μM). (I) Expression of PTX3 were detected by Western blot.

We screened genes that were reported to be associated with CHI3L1 through web-based GWAS analysis, and then selected four genes that were reported to be associated with the inflammatory response: *Ctsd, Ido1, Loxl2*, and *Ptx3* (Fig. 7a, Supplementary Figure S2A). To investigate whether the expression of these genes is related to CHI3L1, we compared the expression levels of these four genes in BV-2 cells with CHI3L1 overexpressing environment and CHI3L1 deficient environment. Expression of all these four genes was increased significantly in CHI3L1 overexpressing environment, but only expression of *Ptx3* was decreased significantly in CHI3L1 deficient environment in BV-2 cells (Supplementary Figure S2B, S2C). The expression of *Ptx3* was elevated by Aβ treatment, and the expression of *Ptx3* was reduced by the treatment of K284-6111 (Fig. 7b). In order to investigate the effect of K284-6111 on the level of PTX3 in the brain of Tg2576 mice and microglial BV-2 cells stimulated by Aβ, we performed Western blot analysis. In the brains of the K284-6111-treated group, the levels of PTX3 were lower than that of the control group (Fig. 7c). In BV-2 cells, the PTX3 level was increased by Aβ treatment, and concentration-dependently decreased by K284-6111 treatment (Fig. 7d). To investigate the correlation between PTX3 and neuroinflammation, BV-2 cells were transfected with PTX3 siRNA and the levels of inflammatory cytokines were determined by qRT-PCR, and inflammation-related proteins were determined by Western blotting. In control siRNA-treated cells, the levels of pro-inflammatory cytokines were significantly increased by Aβ treatment. However, in PTX3-knockdown BV-2 cells treated Aβ showed significantly lower expression levels of M1 microglia markers such as *Tnf*, *Il1b, Il6,* and *Cd86* than normal cells (*n* = 4–6; *Tnf*: F(3, 18) = 38.52, *p* < 0.0001; *Il1b*: F(3, 18) = 247.9, *p* < 0.0001; *Il6*: F(3, 19) = 121.4, *p* < 0.0001; *Cd86*: F(3, 18) = 48.29, *p* = 0.0001) (Fig. 7e). When PTX3 siRNA was treated, the Aβ-induced levels of iNOS, COX-2, and p-IκBα were lower in the siRNA-treated group than in the control group (Fig. 7f, g) but not in p-ERK 1/2. In order to investigate which signaling pathway is involved in the expression of PTX3, the levels of PTX3 were measured by western blot in Aβ-treated BV-2 cells or in CHI3L1 overexpressing BV-2 cells with treatment of ERK inhibitor and NF-kB inhibitor. The level of PTX3 increased when Aβ was treated or when CHI3L1 was overexpressed, and significantly decreased when U0126 and K284-6111 were treated (Fig. 7h, i).

## Discussion

Our previous study suggested that K284-6111 could act as an inhibitor of CHI3L1 by directly binding to CHI3L1 [34]. In this study, we also found that the administration of K284-6111 markedly attenuated impaired cognitive function and memory in the Tg2576 AD mouse model. Consistent with the impaired memory and cognitive mitigating effects, K284-6111 relieved amyloidogenesis and neuroinflammation in Tg2576 mouse. In addition, K284-6111 affected the ERK and NF-κB signaling pathways involved in the neuroinflammation associated with the development and progression of AD.

Neuroinflammation, which includes the activation of microglia and astrocytes, the immune cells of the CNS, is known to contribute to the development of neurodegenerative diseases [36]. Inflammatory responses, including the release of pro-inflammatory cytokines and reactive oxygen species, can damage neurons, leading to synaptic dysfunction or loss and even neuronal death [37]. Activation of immune cells in the brain and increased expression of pro-inflammatory cytokines, i.e., neuroinflammation, is one of the main features of AD [38]. In serum of AD patients, pro-inflammatory cytokines such as TNF-α, IL-1β, and IL-6 have been reported to be higher than normal [39, 40]. Nobili et al. showed that neuroinflammatory events, such as activation of microglia and astrocytes, occurred in Tg2576 mice compared with WT mice [41]. In this study, we showed that administration of K284-6111 resulted in decreased levels of pro-inflammatory cytokines such as TNF-α, IL-1β, and IL-6 in the brain of Tg2576 mice, while simultaneously inhibiting the activation of microglia and astrocytes. In addition, K284-6111 had the effect of inhibiting the expression of Aβ-induced pro-inflammatory cytokines in BV-2 cells. The expression of iNOS and the increase of NO synthesis from iNOS contribute to the pathology of AD, and the increased expression of iNOS in the brains of AD patients has been reported [42]. Nathan et al. reported that iNOS deficiency has a protective effect against the neurotoxicity of Aβ and that iNOS may be considered a major factor in increasing Aβ accumulation [43]. There are studies showing that COX-2 is upregulated in AD patients’ hippocampus and there is a study that COX-2 influences APP processing and accelerates amyloidogenesis in the brain [44, 45]. We found that K284-6111 reduced the levels of iNOS and COX-2 in the brain of Tg2576 mice and inhibited Aβ induced iNOS and COX-2 expressions in BV-2 cells. These data suggest that K284-6111 could be effective for AD treatment through inhibition of neuroinflammation.

CHI3L1 is specifically expressed in diseases involving inflammation, such as inflammatory bowel disease, hepatitis, and asthma. Little is known about how CHI3L1 functions in the inflammatory response, but it plays a critical role in exacerbating the process of inflammation [46]. In the brain, the activated microglia due to neuroinflammation produce CHI3L1 [47]. Sanfilippo et al. discussed that the secretion of CHI3L1 by microglia and astrocytes could lead to peripheral immune cell infiltration including monocyte and macrophage to the brain and consequently, could increase neuronal death [23]. Pranzatelli et al. reported that the CHI3L1 is increased in CSF and serum of inflammatory neurological disorders in children [48]. In our previous study, the Aβ-induced AD mouse model showed a higher level of CHI3L1 in the brain compared with that of controls, and we also demonstrated that CHI3L1-knockdown reduced inflammatory proteins such as iNOS and COX-2 in LPS-stimulated BV-2 cells [34]. The concept of microglia polarization covered in this study is still controversial. Several studies also suggest that microglia polarization does not exist [49]. Our results showed that K284-6111 reduced the M1 markers at Tg2576, whereas it did not affect the M2 markers. Consistent with our previous findings, the present study showed that overexpression of CHI3L1 increased expression of pro-inflammatory cytokines, *Cd86*, one of the markers of M1 microglia, and inflammatory proteins in CHI3L1 overexpressing BV-2 cells. Other studies have reported that CHI3L1 contributes to polarization to M2 macrophage [50–52], but in our results, K284-6111-administrated Tg2576 mouse showed lower mRNA level of *Tnf, Il1b, Il6,* and *Cd86*. Moreover, overexpression of CHI3L1 increased the expression of markers of M1 microglia, *Tnf, Il1b, Il6,* and *Cd86*, and inhibited by K284-6111 treatment.

ERK and NF-κB signals are known to be highly involved in AD and neuroinflammation. There have been numerous studies on the relationship between increased Aβ level and activation of ERK pathway, suggesting that ERK signal could be related to AD [53–55]. Chronic activation of ERK pathway was found in the hippocampus slide of the Aβ overexpressing AD transgenic animal model [53]; moreover, Kirouac et al. reported that the level of p-ERK in AD patients’ brain increased as AD progressed [54]. Park et al. showed that Asiatic acid inhibited methamphetamine-induced neuroinflammation through blocking of ERK pathway [55]. NF-κB could modulate more than 400 different genes including genes engaged in innate immunity and associated with AD [56]. Chen et al. demonstrated that BACE1, which is deeply involved in amyloidogenesis, was upregulated when the NF-κB signal was activated [57]. Studies have been conducted on drugs that have been effective in alleviating AD and neuroinflammation by inhibiting the NF-κB signal. Alawdi et al. reported that nanodiamond could exerts neuroprotective effect in AD rat model through modulating the NF-κB signal [58]. In our previous study, bee venom, ethanol extract of *Nannochloropsis oceanica*, and punicalagin had an inhibitory effect on neuroinflammation and amyloidogenesis through blockade of NF-κB pathway [59–61]. Based on our in vivo and in vitro results, K284-6111 inhibits NF-κB and ERK signaling. The reduction of p-IκBα and of p-ERK in the brain of Tg2576 mice administered K284-6111 was greater than that of p-JNK or p-p38. And we observed that when K284-6111 was treated in Aβ-induced BV-2 cells, only p-IκBα and p-ERK decreased in a concentration-dependent manner. We found that ERK and NF-κB signals were activated in BV-2 cells with increased CHI3L1 expression. The ERK and NF-κB signals activated by CHI3L1 overexpression were inhibited by the treatment of K284-6111. Consistent with our findings, Tang et al. showed that ERK and NF-κB signals were activated in a concentrate-dependent manner when recombinant CHI3L1 (YKL-40) was treated to Beas-2B cells [62]. He et al. demonstrated that CHI3L1 binds to IL-13Rα2 and induces ERK, AKT, and Wnt/β-catenin signals independent of IL-13 pathway [63]. Subramaniam et al. described in their review paper that CHI3L1 binds to RAGE (receptor for advance glycation end product), resulting in the activation of the NF-κB, β-catenin, and MAPK signaling pathways [64]. In addition, we observed that p-IκBα was inhibited when the ERK inhibitor was treated; however, p-ERK was not changed when the NF-κB inhibitor was treated. Thus, the ERK-dependent NF-κB pathway could be associated with reduced neuroinflammation by K284-6111.

**Fig. 8:**
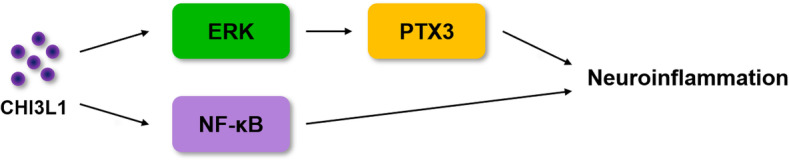
CHI3L1 mediates neuroinflammation through NF-κB pathway and ERK dependent PTX3 pathway

PTX3 is also known to be involved in the amplification of the inflammatory response and innate immune regulation, and therefore it could be a candidate marker for inflammation in many chronic diseases [65]. We found that PTX3 is associated with CHI3L1 through the web-based GWAS analysis; moreover, we verified the association between CHI3L1 and PTX3 experimentally in BV-2 cells. The expression of PTX3 was increased when CHI3L1 expression increased, and the expression of PTX3 was decreased when CHI3L1 knock-downed. We observed that when PTX3 was knockdown, Aβ-induced iNOS, COX-2, and p-IκBα except p-ERK were decreased. We also observed that when PTX3 was knockdown, Aβ-induced M1 microglia markers expressions including *Tnf, Il1b, Il6,* and *Cd86* were decreased. In addition, we found that K284-6111 inhibits ERK and NF-κB signaling by inhibiting CHI3L1. Several previous studies reported that PTX3 could be regulated by ERK signals and that PTX3 could regulate NF-κB signals. Hwang et al. reported that the elevated PTX3 expression by sodium iodate treatment was decreased when treated with ERK inhibitors, in primary human H-RPE and ARPE-19 cells [66]. Also, Zhang et al. showed that JNK and ERK specific inhibitors downregulate TNF-induced PTX3 promoter activity and PTX3 release in HASMC cells [65]. Qi et al. reported that the silencing of PTX3 mitigates the LPS-induced inflammatory response in BV-2 cells and mice, which occurs by down-regulating the TLR4/NF-κB signaling pathway [67]. Ahmmed et al. showed when they caused the deglycosylation of PTX3 and changed its function, the AKT/NF-κB signaling pathway was inactivated [68]. These data indicated that PTX3 pathway could play a significant role in K284-6111 inhibiting effect on CHI3L1-mediating M1-specific neuroinflammation associated with AD development (Fig. 8).

In this study, the roles of K284-6111 and CHI3L1 in terms of neuroinflammation were addressed, but in terms of neurogenesis and death were not addressed. To find out the roles of CHI3L1 in AD, there is a need for further study in this regard. Our current study demonstrated that CHI3L1 is an important factor for AD pathogenesis and neuroinflammation and suggests the possibility of K284-6111, an inhibitor of CHI3L1, as a new therapeutic candidate for AD patients.

## Conclusion

These results suggest that CHI3L1 exacerbate neuroinflammation through ERK-mediated PTX3 and NF-κB pathways, and become a new therapeutic target for AD. Therefore, the CHI3L1 inhibitor, K284-6111, is a potential candidate as a therapeutic agent that could relieve neuroinflammation and could improve memory dysfunction.

## Supplementary Information

**Additional file 1: Table S1.** List and sequences of qPCR primers for mRNA expression. **Figure S1.** The CHI3L1 levels (A) in serum and (B) in brain were assessed using the specific ELISA kits. **Figure S2.** PTX3 is associated with CHI3L1 (A) Gene network analysis associated with CHI3L1 was carried out using the web-based analysis tool. The mRNA expression level of *Chi3l1, Cd163, Ctsd, Ido1*, and *Ptx3* were assessed by qRT-PCR. (B) BV-2 cells were transfected with CHI3L1-expression vector. (C) BV-2 cells were transfected with CHI3L1 siRNA (40 nM).

## Data Availability

The datasets generated and/or analyzed during the current study are available from the corresponding author on reasonable request.

## Abbreviations

Aβ: β-amyloid
AD: Alzheimer’s disease
APP: Amyloid precursor protein
Arg1: Arginase-1
BACE1: β-secretase 1
CHI3L1: Chitinase-3-like 1
CNS: Central nervous system
COX-2: Cyclooxygenase 2
ERK: Extracellular signal-regulated kinases
GFAP: Glial fibrillary acidic protein
IBA-1: Ionized calcium-binding adaptor molecule 1
IκB: Nuclear factor-kappa B inhibitor
IL: Interleukin
iNOS: Inducible nitric oxide synthase
LPS: Lipopolysaccharide
MRC1: Mannose receptor C-type 1
NF-κB: Nuclear factor-kappa B
PTX3: Pentraxin-3
TGF: Transforming growth factor
TNF: tumor necrosis factor

## References

[1] Khoury R, Patel K, Gold J, Hinds S, Grossberg GT (2017). Recent progress in the pharmacotherapy of Alzheimer's disease. Drugs & Aging.

[2] Mangold CA, Szpara ML. Persistent Infection with Herpes Simplex Virus 1 and Alzheimer's Disease-A Call to Study How Variability in Both Virus and Host may Impact Disease. Viruses. 2019:11(10). 10.3390/v11100966 PMC6833100 31635156

[3] Nazem A, Sankowski R, Bacher M, Al-Abed Y (2015). Rodent models of neuroinflammation for Alzheimer's disease. Journal of Neuroinflammation.

[4] Ham HJ, Han SB, Yun J, Yeo IJ, Ham YW, Kim SH, Park PH, Choi DY, Hong JT (2019). Bee venom phospholipase A2 ameliorates amyloidogenesis and neuroinflammation through inhibition of signal transducer and activator of transcription-3 pathway in Tg2576 mice. Translational Neurodegeneration.

[5] Liu PP, Xie Y, Meng XY, Kang JS (2019). History and progress of hypotheses and clinical trials for Alzheimer's disease. Signal Transduction and Targeted Therapy.

[6] Webers A, Heneka MT, Gleeson PA. The role of innate immune responses and neuroinflammation in amyloid accumulation and progression of Alzheimer's disease. Immunology and cell biology. 2019. 10.1111/imcb.12301 31654430

[7] Wes PD, Sayed FA, Bard F, Gan L (2016). Targeting microglia for the treatment of Alzheimer's Disease. Glia.

[8] Tang Y, Le W (2016). Differential Roles of M1 and M2 Microglia in neurodegenerative diseases. Molecular Neurobiology.

[9] Yang X, Xu S, Qian Y, Xiao Q (2017). Resveratrol regulates microglia M1/M2 polarization via PGC-1alpha in conditions of neuroinflammatory injury. Brain, Behavior, and Immunity.

[10] McQuade A, Blurton-Jones M (2019). Microglia in Alzheimer's Disease: exploring how genetics and phenotype influence risk. Journal of Molecular Biology.

[11] Zhou T, Huang Z, Sun X, Zhu X, Zhou L, Li M, Cheng B, Liu X, He C (2017). Microglia Polarization with M1/M2 Phenotype Changes in rd1 Mouse Model of Retinal Degeneration. Frontiers in Neuroanatomy.

[12] Peng H, Geil Nickell CR, Chen KY, McClain JA, Nixon K (2017). Increased expression of M1 and M2 phenotypic markers in isolated microglia after four-day binge alcohol exposure in male rats. Alcohol.

[13] Yao K, Zu HB. Microglial polarization: novel therapeutic mechanism against Alzheimer's disease. Inflammopharmacology. 2019. 10.1007/s10787-019-00613-5 31264132

[14] Sarlus H, Heneka MT (2017). Microglia in Alzheimer's disease. The Journal of Clinical Investigation.

[15] Komi DE, Kazemi T, Bussink AP (2016). New insights into the relationship between Chitinase-3-Like-1 and asthma. Current Allergy and Asthma Reports.

[16] Lee DH, Kim KC, Hwang CJ, Park KR, Jung YS, Kim SY, Kim JY, Song JK, Song MJ, Choi MK (2019). Decreased lung tumor development in SwAPP mice through the downregulation of CHI3L1 and STAT 3 activity via the upregulation of miRNA342-3p. Molecular therapy Nucleic acids.

[17] Kim KC, Yun J, Son DJ, Kim JY, Jung JK, Choi JS, Kim YR, Song JK, Kim SY, Kang SK (2018). Suppression of metastasis through inhibition of chitinase 3-like 1 expression by miR-125a-3p-mediated up-regulation of USF1. Theranostics.

[18] Salomon J, Matusiak L, Nowicka-Suszko D, Szepietowski JC (2017). Chitinase-3-Like Protein 1 (YKL-40) Is a New Biomarker of Inflammation in Psoriasis. Mediators of Inflammation.

[19] Querol-Vilaseca M, Colom-Cadena M, Pegueroles J, San Martin-Paniello C, Clarimon J, Belbin O, Fortea J, Lleo A (2017). YKL-40 (Chitinase 3-like I) is expressed in a subset of astrocytes in Alzheimer's disease and other tauopathies. Journal of Neuroinflammation.

[20] Coffman FD (2008). Chitinase 3-Like-1 (CHI3L1): a putative disease marker at the interface of proteomics and glycomics. Critical Reviews in Clinical Laboratory Sciences.

[21] Libreros S, Garcia-Areas R, Shibata Y, Carrio R, Torroella-Kouri M, Iragavarapu-Charyulu V (2012). Induction of proinflammatory mediators by CHI3L1 is reduced by chitin treatment: decreased tumor metastasis in a breast cancer model. International Journal of Cancer.

[22] Di Rosa M, Malaguarnera L (2016). Chitinase 3 Like-1: an emerging molecule involved in diabetes and diabetic complications. Pathobiology.

[23] Sanfilippo C, Longo A, Lazzara F, Cambria D, Distefano G, Palumbo M, Cantarella A, Malaguarnera L, Di Rosa M (2017). CHI3L1 and CHI3L2 overexpression in motor cortex and spinal cord of sALS patients. Molecular and Cellular Neurosciences.

[24] Dai QH, Gong DK (2019). Association of the polymorphisms and plasma Level of CHI3L1 with Alzheimer's disease in the chinese han population: a case-control study. Neuropsychobiology.

[25] Bonneh-Barkay D, Wang G, Starkey A, Hamilton RL, Wiley CA (2010). In vivo CHI3L1 (YKL-40) expression in astrocytes in acute and chronic neurological diseases. Journal of Neuroinflammation.

[26] Salomon J, Matusiak L, Nowicka-Suszko D, Szepietowski JC (2017). Chitinase-3-Like Protein 1 (YKL-40) Reflects the Severity of Symptoms in Atopic Dermatitis. Journal of Immunology Research.

[27] Libreros S, Garcia-Areas R, Iragavarapu-Charyulu V (2013). CHI3L1 plays a role in cancer through enhanced production of pro-inflammatory/pro-tumorigenic and angiogenic factors. Immunologic Research.

[28] Chen F, An Y, Wang J (2017). CHI3L1 is correlated with childhood asthma. Int J Clin Exp Pathol.

[29] Muszynski P, Groblewska M, Kulczynska-Przybik A, Kulakowska A, Mroczko B (2017). YKL-40 as a Potential biomarker and a possible target in therapeutic strategies of Alzheimer's disease. Current Neuropharmacology.

[30] Soeki T, Bando S, Uematsu E, Matsuura T, Niki T, Ise T, Kusunose K, Hotchi J, Ueda Y, Tomita N (2014). Pentraxin 3 is a local inflammatory marker in atrial fibrillation. Heart and vessels.

[31] Luchetti MM, Piccinini G, Mantovani A, Peri G, Matteucci C, Pomponio G, Fratini M, Fraticelli P, Sambo P, Di Loreto C (2000). Expression and production of the long pentraxin PTX3 in rheumatoid arthritis (RA). Clinical and experimental immunology.

[32] Zhao Y, Feng G, Wang Y, Yue Y, Zhao W (2014). A key mediator, PTX3, of IKK/IkappaB/NF-kappaB exacerbates human umbilical vein endothelial cell injury and dysfunction. International journal of clinical and experimental pathology.

[33] Ko CY, Chang LH, Lee YC, Sterneck E, Cheng CP, Chen SH, Huang AM, Tseng JT, Wang JM (2012). CCAAT/enhancer binding protein delta (CEBPD) elevating PTX3 expression inhibits macrophage-mediated phagocytosis of dying neuron cells. Neurobiology of aging.

[34] Choi JY, Yeo IJ, Kim KC, Choi WR, Jung JK, Han SB, Hong JT (2018). K284-6111 prevents the amyloid beta-induced neuroinflammation and impairment of recognition memory through inhibition of NF-kappaB-mediated CHI3L1 expression. Journal of neuroinflammation.

[35] Morris R (1984). Developments of a water-maze procedure for studying spatial learning in the rat. Journal of neuroscience methods.

[36] Niranjan R (2018). Recent advances in the mechanisms of neuroinflammation and their roles in neurodegeneration. Neurochemistry international.

[37] Schain M, Kreisl WC (2017). Neuroinflammation in Neurodegenerative Disorders-a Review. Current neurology and neuroscience reports.

[38] Su F, Bai F, Zhang Z (2016). Inflammatory cytokines and Alzheimer's Disease: a review from the perspective of genetic polymorphisms. Neuroscience Bulletin.

[39] Domingues C, da Cruz ESOAB, Henriques AG (2017). Impact of Cytokines and Chemokines on Alzheimer's Disease Neuropathological Hallmarks. Current Alzheimer Research.

[40] Khemka VK, Ganguly A, Bagchi D, Ghosh A, Bir A, Biswas A, Chattopadhyay S, Chakrabarti S (2014). Raised serum proinflammatory cytokines in Alzheimer's disease with depression. Aging and Disease.

[41] Nobili A, Latagliata EC, Viscomi MT, Cavallucci V, Cutuli D, Giacovazzo G, Krashia P, Rizzo FR, Marino R, Federici M (2017). Dopamine neuronal loss contributes to memory and reward dysfunction in a model of Alzheimer's disease. Nature Communications.

[42] Medeiros R, Prediger RD, Passos GF, Pandolfo P, Duarte FS, Franco JL, Dafre AL, Di Giunta G, Figueiredo CP, Takahashi RN (2007). Connecting TNF-alpha signaling pathways to iNOS expression in a mouse model of Alzheimer's disease: relevance for the behavioral and synaptic deficits induced by amyloid beta protein. J Neurosci.

[43] Nathan C, Calingasan N, Nezezon J, Ding A, Lucia MS, La Perle K, Fuortes M, Lin M, Ehrt S, Kwon NS (2005). Protection from Alzheimer's-like disease in the mouse by genetic ablation of inducible nitric oxide synthase. The Journal of Experimental Medicine.

[44] O'Banion MK (1999). COX-2 and Alzheimer's disease: potential roles in inflammation and neurodegeneration. Expert Opinion on Investigational Drugs.

[45] Xiang Z, Ho L, Yemul S, Zhao Z, Qing W, Pompl P, Kelley K, Dang A, Qing W, Teplow D (2002). Cyclooxygenase-2 promotes amyloid plaque deposition in a mouse model of Alzheimer's disease neuropathology. Gene Expression.

[46] Eurich K, Segawa M, Toei-Shimizu S, Mizoguchi E (2009). Potential role of chitinase 3-like-1 in inflammation-associated carcinogenic changes of epithelial cells. World Journal of Gastroenterology.

[47] Yeo IJ, Lee CK, Han SB, Yun J, Hong JT (2019). Roles of chitinase 3-like 1 in the development of cancer, neurodegenerative diseases, and inflammatory diseases. Pharmacology & Therapeutics.

[48] Pranzatelli MR, Tate ED, McGee NR (2017). Microglial/macrophage markers CHI3L1, sCD14, and sCD163 in CSF and serum of pediatric inflammatory and non-inflammatory neurological disorders: A case-control study and reference ranges. Journal of the Neurological Sciences.

[49] Ransohoff RM (2016). A polarizing question: do M1 and M2 microglia exist?. Nature neuroscience.

[50] Steenbrugge J, Breyne K, Demeyere K, De Wever O, Sanders NN, Van Den Broeck W, Colpaert C, Vermeulen P, Van Laere S, Meyer E (2018). Anti-inflammatory signaling by mammary tumor cells mediates prometastatic macrophage polarization in an innovative intraductal mouse model for triple-negative breast cancer. J Exp Clin Cancer Res.

[51] Jingjing Z, Nan Z, Wei W, Qinghe G, Weijuan W, Peng W, Xiangpeng W (2017). MicroRNA-24 Modulates Staphylococcus aureus-Induced Macrophage Polarization by Suppressing CHI3L1. Inflammation.

[52] Xu N, Bo Q, Shao R, Liang J, Zhai Y, Yang S, Wang F, Sun X (2019). Chitinase-3-Like-1 promotes M2 macrophage differentiation and induces choroidal neovascularization in neovascular age-related macular degeneration. Investigative Ophthalmology & Visual Science.

[53] Rai SN, Dilnashin H, Birla H, Singh SS, Zahra W, Rathore AS, Singh BK, Singh SP (2019). The Role of PI3K/Akt and ERK in Neurodegenerative Disorders. Neurotoxicity Research.

[54] Kirouac L, Rajic AJ, Cribbs DH, Padmanabhan J. Activation of Ras-ERK signaling and GSK-3 by amyloid precursor protein and amyloid beta facilitates neurodegeneration in Alzheimer's disease. eNeuro. 2017:4(2). 10.1523/ENEURO.0149-16.2017 PMC5367084 28374012

[55] Park JH, Seo YH, Jang JH, Jeong CH, Lee S, Park B (2017). Asiatic acid attenuates methamphetamine-induced neuroinflammation and neurotoxicity through blocking of NF-kB/STAT3/ERK and mitochondria-mediated apoptosis pathway. Journal of Neuroinflammation.

[56] Jha NK, Jha SK, Kar R, Nand P, Swati K, Goswami VK (2019). Nuclear factor-kappa beta as a therapeutic target for Alzheimer's disease. Journal of Neurochemistry.

[57] Chen CH, Zhou W, Liu S, Deng Y, Cai F, Tone M, Tone Y, Tong Y, Song W (2012). Increased NF-kappaB signalling up-regulates BACE1 expression and its therapeutic potential in Alzheimer's disease. Int J Neuropsychopharmacol.

[58] Alawdi SH, El-Denshary ES, Safar MM, Eidi H, David MO, Abdel-Wahhab MA (2017). Neuroprotective Effect of Nanodiamond in Alzheimer's Disease Rat Model: a Pivotal Role for Modulating NF-kappaB and STAT3 Signaling. Molecular Neurobiology.

[59] Choi JY, Hwang CJ, Lee HP, Kim HS, Han SB, Hong JT (2017). Inhibitory effect of ethanol extract of Nannochloropsis oceanica on lipopolysaccharide-induced neuroinflammation, oxidative stress, amyloidogenesis and memory impairment. Oncotarget.

[60] Gu SM, Park MH, Hwang CJ, Song HS, Lee US, Han SB, Oh KW, Ham YW, Song MJ, Son DJ (2015). Bee venom ameliorates lipopolysaccharide-induced memory loss by preventing NF-kappaB pathway. Journal of Neuroinflammation.

[61] Kim YE, Hwang CJ, Lee HP, Kim CS, Son DJ, Ham YW, Hellstrom M, Han SB, Kim HS, Park EK (2017). Inhibitory effect of punicalagin on lipopolysaccharide-induced neuroinflammation, oxidative stress and memory impairment via inhibition of nuclear factor-kappaB. Neuropharmacology.

[62] Tang H, Sun Y, Shi Z, Huang H, Fang Z, Chen J, Xiu Q, Li B (2013). YKL-40 induces IL-8 expression from bronchial epithelium via MAPK (JNK and ERK) and NF-kappaB pathways, causing bronchial smooth muscle proliferation and migration. Journal of Immunology.

[63] He CH, Lee CG, Dela Cruz CS, Lee CM, Zhou Y, Ahangari F, Ma B, Herzog EL, Rosenberg SA, Li Y (2013). Chitinase 3-like 1 regulates cellular and tissue responses via IL-13 receptor alpha2. Cell Reports.

[64] Subramaniam R, Mizoguchi A, Mizoguchi E (2016). Mechanistic roles of epithelial and immune cell signaling during the development of colitis-associated cancer. Cancer Research Frontiers.

[65] Zhang J, Koussih L, Shan L, Halayko AJ, Chen BK, Gounni AS (2015). TNF up-regulates Pentraxin3 expression in human airway smooth muscle cells via JNK and ERK1/2 MAPK pathways. Allergy, Asthma & Clinical Immunology.

[66] Hwang N, Kwon MY, Woo JM, Chung SW. Oxidative Stress-induced pentraxin 3 expression human retinal pigment epithelial cells is involved in the pathogenesis of age-related macular degeneration. Int J Mol Sci. 2019;20(23). 10.3390/ijms20236028 PMC6928709 31795454

[67] Qi S, Zhao F, Li Z, Liang F, Yu S. Silencing of PTX3 alleviates LPS-induced inflammatory pain by regulating TLR4/NF-kappaB signaling pathway in mice. Bioscience Reports. 2020:40(2). 10.1042/BSR20194208 PMC7000368 31957804

[68] Ahmmed B, Khan MN, Nisar MA, Kampo S, Zheng Q, Li Y, Yan Q (2019). Tunicamycin enhances the suppressive effects of cisplatin on lung cancer growth through PTX3 glycosylation via AKT/NF-kappaB signaling pathway. International Journal of Oncology.

