# Supplementary material for: K284-6111 alleviates memory impairment and neuroinflammation in Tg2576 mice by inhibition of Chitinase-3-like 1 regulating ERK-dependent PTX3 pathway
Source: J Neuroinflammation. 2020 Nov 22;17:350. doi: 10.1186/s12974-020-02022-w (PMC7681957; doi:10.1186/s12974-020-02022-w)
Supplement: Supplementary file 1 — Additional file 1: Table S1. List and sequences of qPCR primers for mRNA expression. Figure S1. The CHI3L1 levels (A) in serum and (B) in brain were assessed using the specific ELISA kits. Figure S2. PTX3 is associated with CHI3L1 (A) Gene network analysis associated with CHI3L1 was carried out using the web-based analysis tool. The mRNA expression level of Chi3l1, Cd163, Ctsd, Ido1, and Ptx3 were assessed by qRT-PCR. (B) BV-2 cells were transfected with CHI3L1-expression vector. (C) BV-2 cells were transfected with CHI3L1 siRNA (40 nM). [file 12974_2020_2022_MOESM1_ESM.docx]

**Supplementary information for**

**K284-6111 alleviates memory impairment and neuroinflammation in Tg2576 mice by inhibition of Chitinase-3-like 1 regulating ERK dependent PTX3 pathway**

Hyeon Joo Ham^1^, Yong Sun Lee^1^, Jaesuk Yun^1^, Dong Ju Son^1^, Hee Pom Lee^1^, Sang-Bae Han^1^, and Jin Tae Hong^1^

^1^College of Pharmacy and Medical Research Center, Chungbuk National University, Osongsaengmyeong 1-ro, Osong-eup, Heungdeok-gu, Cheongju, Chungbuk, 28160, Republic of Korea

*Correspondence: Dr. Jin Tae Hong (jinthong@chungbuk.ac.kr), College of Pharmacy and Medical Research Center, Chungbuk National University, Osongsaengmyeong 1-ro, Osong-eup, Heungdeok-gu, Cheongju, Chungbuk, 28160, Republic of Korea, Tel: +82-043-261-2813, Fax: +82-043-268-2732.

Hyeon Joo Ham: [prodijoo0918@nate.com](mailto:prodijoo0918@nate.com)

Yong Sun Lee: [kallintz@gmail.com](mailto:kallintz@gmail.com)

Jaesuk Yun: [jyun@chungbuk.ac.kr](mailto:jyun@chungbuk.ac.kr)

Dong Ju Son: [sondj1@chungbuk.ac.kr](mailto:sondj1@chungbuk.ac.kr)

Hee Pom Lee: [heepom@empas.com](mailto:heepom@empas.com)

Sang-Bae Han: [shan@chungbuk.ac.kr](mailto:shan@chungbuk.ac.kr)

**Supplementary Table S1. List and sequences of qPCR primers for mRNA expression**

|  | **Forward (5’→3’)** | **Reverse (5’→3’)** | **Species** |
| --- | --- | --- | --- |
| ***β-actin*** | GGCTGTATTCCCCTCCATCG | CCAGTTGGTAACAATGCCATGT | Mouse |
| ***Tnf*** | TCTTCTCATTCCTGCTTGTGG | CACTTGGTGGTTTGCTACGA | Mouse |
| ***Il1b*** | CCTTCCAGGATGAGGACATGA | TGAGTCACAGAGGATGGGCTC | Mouse |
| ***Il6*** | GAGGATACCACTCCCAACAGACC | AAGTGCATCATCGTTGTTCATACA | Mouse |
| ***Chi3l1*** | GTACAAGCTGGTCTGCTACTTC | ATGTGCTAAGCATGTTGTCGC | Mouse |
| ***Cd86*** | TGTTTCCGTGGAGACGCAAG | TTGAGCCTTTGTAAATGGGCA | Mouse |
| ***Arg1*** | CTCCAAGCCAAAGTCCTTAGAG | AGGAGCTGTCATTAGGGACATC | Mouse |
| ***Mrc1*** | CTCTGTTCAGCTATTGGACGC | CGGAATTTCTGGGATTCAGCTTC | Mouse |
| ***Tgfb*** | CTCCCGTGGCTTCTAGTGC | GCCTTAGTTTGGACAGGATCTG | Mouse |
| ***Il10*** | GCTCTTACTGACTGGCATGAG | CGCAGCTCTAGGAGCATGTG | Mouse |
| ***Ptx3*** | CCTGCGATCCTGCTTTGTG | GGTGGGATGAAGTCCATTGTC | Mouse |
| ***Cd163*** | ATGGGTGGACACAGAATGGTT | CAGGAGCGTTAGTGACAGCAG | Mouse |
| ***Ctsd*** | CCTGGCTTCGTCCTCCTTC | GGCGATGACTGCATGGAGT | Mouse |
| ***Ido1*** | GCTTTGCTCTACCACATCCAC | CAGGCGCTGTAACCTGTGT | Mouse |

**Supplementary figure S1.**

**
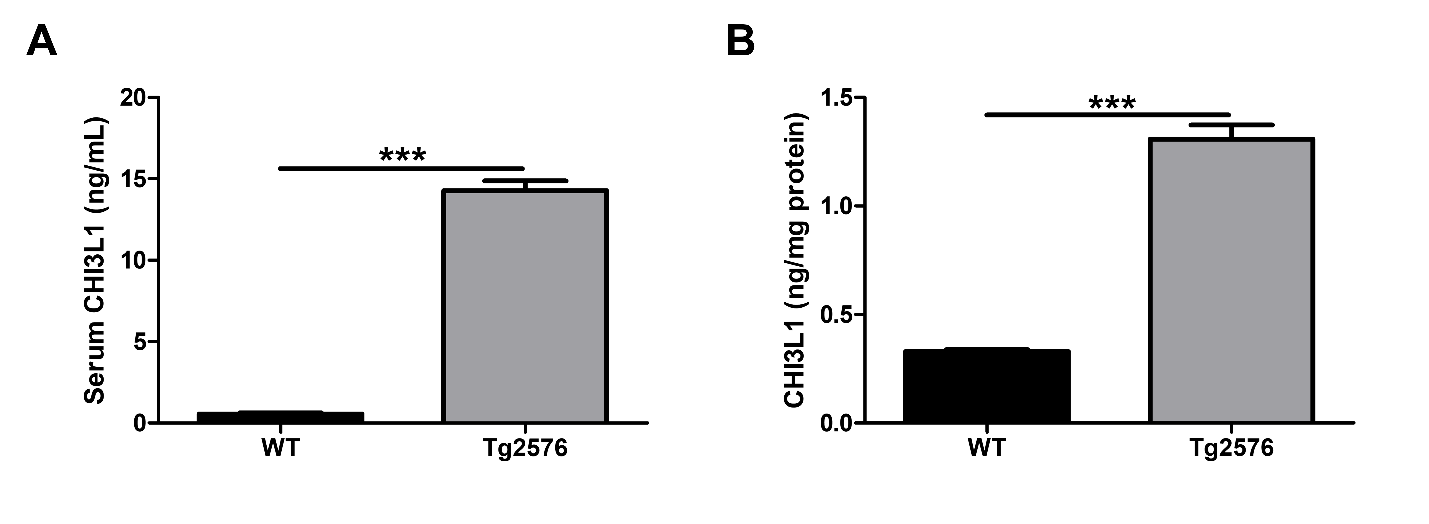
**

The CHI3L1 levels (A) in serum and (B) in brain were assessed using the specific ELISA kits.

**Supplementary figure S2. PTX3 is associated with CHI3L1**

**
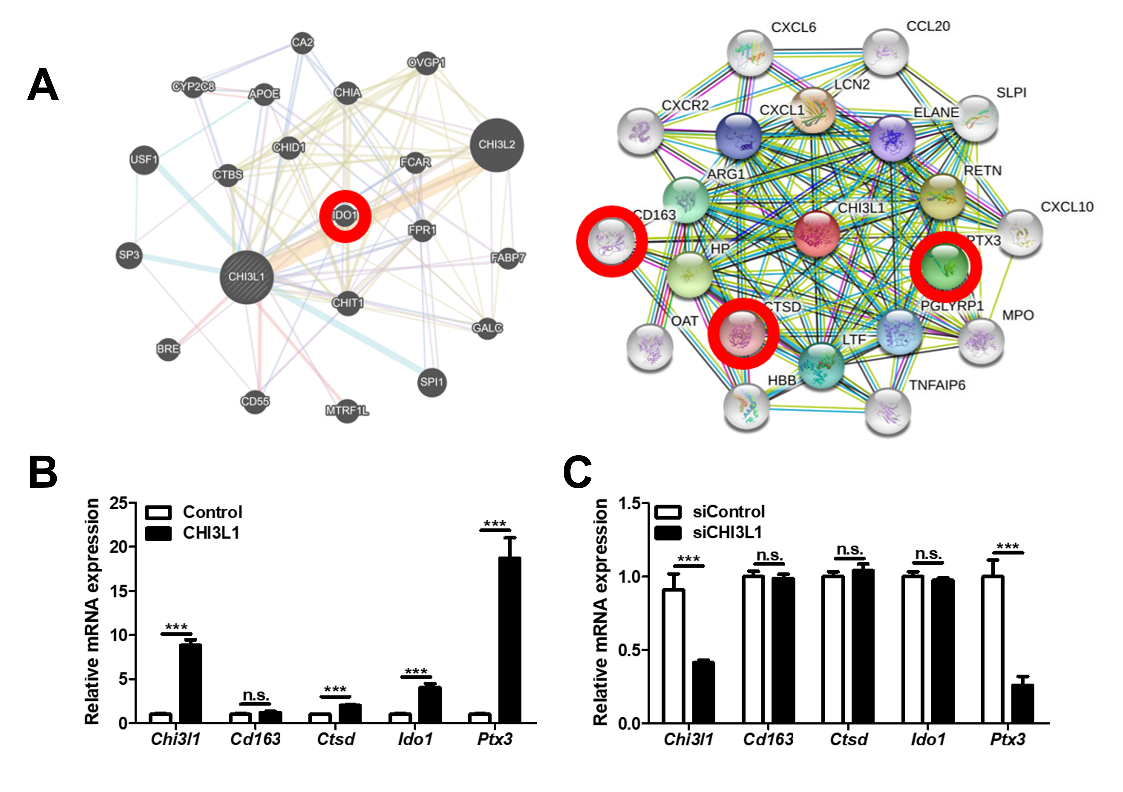
**

(A) Gene network analysis associated with CHI3L1 was carried out using the web-based analysis tool. The mRNA expression level of *Chi3l1, Cd163, Ctsd, Ido1*, and *Ptx3* were assessed by qRT-PCR. (B) BV-2 cells were transfected with CHI3L1-expression vector. (C) BV-2 cells were transfected with CHI3L1 siRNA (40 nM).
